# A Step-by-Step Guide for the Novel Radiometal Production for Medical Applications: Case Studies with ^68^Ga, ^44^Sc, ^177^Lu and ^161^Tb

**DOI:** 10.3390/molecules25040966

**Published:** 2020-02-20

**Authors:** Zeynep Talip, Chiara Favaretto, Susanne Geistlich, Nicholas P. van der Meulen

**Affiliations:** 1 Center for Radiopharmaceutical Sciences ETH-PSI-USZ, Paul Scherrer Institute, 5232 Villigen-PSI, Switzerland; 2 Department of Chemistry and Applied Biosciences, ETH Zurich, 8093 Zurich, Switzerland; 3 Laboratory of Radiochemistry, Paul Scherrer Institute, 5232 Villigen-PSI, Switzerland

**Keywords:** cyclotron, reactor, novel radionuclides, radiopharmaceuticals, imaging, therapy, theranostics, scandium-44, terbium-161, gallium-68, lutetium-177, Good Manufacturing Practice

## Abstract

The production of novel radionuclides is the first step towards the development of new effective radiopharmaceuticals, and the quality thereof directly affects the preclinical and clinical phases. In this review, novel radiometal production for medical applications is briefly elucidated. The production status of the imaging nuclide ^44^Sc and the therapeutic β^-^-emitter nuclide ^161^Tb are compared to their more established counterparts, ^68^Ga and ^177^Lu according to their targetry, irradiation process, radiochemistry, and quality control aspects. The detailed discussion of these significant issues will help towards the future introduction of these promising radionuclides into drug manufacture for clinical application under Good Manufacturing Practice (GMP).

## 1. Introduction

Cancer is regarded as one of the main health problems faced in the world today. Recent reports show that more than 10.000 hospitals worldwide use radionuclides for in vivo diagnosis or therapy and about 35 million patients receive cancer therapy using radionuclides annually [1]. Other than cancer, radionuclides are used in medicine for the diagnosis and treatment of various diseases, such as cardiovascular and brain disorders. It is expected that the demand for medically-applied radionuclides will dramatically increase in the years to come. According to the recent Fior Markets report, the global radiopharmaceuticals/nuclear medicine market is expected to grow from USD 6.5 billion in 2017 to USD 12.41 billion by 2025 [2].

Different radionuclides are applied in various fields of nuclear medicine. Taking the radionuclide in question’s half-life and decay emission into consideration, they are used for imaging, *via* positron emission tomography (PET) or single-photon emission computed tomography (SPECT), and for therapy *via* α-, β^−^-, or conversion and/or Auger electron emission. Beta particles, alpha particles, and Auger electrons can irradiate tissue volumes with multicellular, cellular and subcellular dimensions, respectively [3,4] thanks to the different linear energy transfer (LET), defined as the amount of energy transferred by a traveling particle to the surrounding material per unit of length. In particular, it is of great interest to develop clinical methods using conversion/Auger-electron-emitting radionuclides towards the treatment of disseminated tumor cells and small metastases. To date, clinical research in this regard has been very limited and there is still much to learn about the Auger electrons as a potential therapeutic [3,4,5,6,7].

Radiometals have been used extensively in clinical diagnostics over the last three decades, particularly, ^99m^Tc, ^67^Ga and ^111^In for SPECT and, more recently, ^68^Ga, ^89^Zr and ^64^Cu for PET (Table 1). On the other hand,, therapeutic radiometals in clinical use are limited to ^177^Lu [8,9,10], ^90^Y [11,12], ^89^Sr [13,14] and ^223^Ra [15,16]. ^223^Ra is the only approved radionuclide by health authorities for targeted alpha therapy to extend survival [17]. It is worth mentioning that to bring a novel radionuclide from the development stage to the clinic is a long process. Phase III data for ^177^Lu demonstrated that radioactive drugs took more than 15 years to achieve the European Medicines Agency (*EMA*) and US Food and Drug Administration (FDA) approval [18]. So far, only ^177^Lu-DOTATATE has been approved for clinical use, while four other ^177^Lu-based radiopharmaceuticals have been clinically tested [19,20,21].

In the last decade, terbium and scandium radionuclides have received special attention for their potential use in radiopharmacy. The therapeutic β^-^-emitter ^161^Tb and the diagnostic radionuclide ^44^Sc are considered as an alternative to ^177^Lu and ^68^Ga, respectively. ^161^Tb can be prepared in high specific activity and it has lower energy γ-emission (48.9 keV (17%), 74.6 keV (10.2%)) than its ^177^Lu counterpart. Dose calculations have shown that ^161^Tb can be more effective for very small tumor lesions due to the emission of low-energy conversion and Auger electrons [23,24]. Moreover, preclinical in-vivo and in-vitro comparison studies with DOTA-folate conjugate and prostate-specific membrane antigen (PSMA)-617 labeled ^177^Lu and ^161^Tb have shown better results for ^161^Tb [25,26,27]. Marin has reported that the SPECT images of ^161^Tb are comparable to those of ^177^Lu using particular settings of the SPECT device in question [28]. Another attractive property for terbium is that, other than ^161^Tb, it has two diagnostic radioisotopes ^152^Tb (T_1/2_ = 17.5 h, Eβ^+^_av_: 1140 keV) for PET and ^155^Tb (T_1/2_ = 5.32 d, Eγ: 87 keV (32%), 105 keV (25%)) for SPECT, making it ideal for the “matched pair” principle of theranostics [29,30].

^44^Sc has promising decay characteristics (T_1/2_ = 4.04 h, 94.27% β^+^ and 5.73% EC, Eβ^+^_av_: 632 keV), which makes it a good alternative to the short-lived ^68^Ga (T_½_ = 67.71 min, 89% β^+^ and 11% EC, Eβ^+^_av_: 830 keV). Since the chemistry of ^44^Sc is similar to those of the lanthanides, it is proposed to be used as a diagnostic match to ^177^Lu and for pre-therapeutic dosimetry studies. Moreover, ^44^Sc has a theranostic β^−^- emitting matched pair ^47^Sc (T_1/2_ = 3.35 d), which is particularly interesting for radionuclide therapy [31,32,33]. Furthermore, the differences between the coordination chemistry of ^68^Ga and ^177^Lu cause deviation in pharmacokinetic studies [34,35]. ^44^Sc has better spatial resolution for PET imaging than ^68^Ga, thanks to its lower positron energy [36]. In addition, its longer half-life (T_1/2_ = 4.04 h) [37] allows performing late-time-point PET imaging and its transfer to PET centers, while ^68^Ga only allows image-taking over a few hours.

This review is divided into two parts: part one briefly covers the required steps for the production of novel radionuclides towards medical application, while part two outlines the status of the production of two emerging novel radionuclides, namely, ^44^Sc and ^161^Tb. Their progress is explained in detail in comparison with the clinically-applied radionuclides, ^68^Ga and ^177^Lu, respectively.

## 2. Radionuclide Production: A Step-by-Step Guide

The production of novel radionuclides for innovative radiopharmaceuticals requires multidisciplinary collaboration between nuclear physicists, material scientists, radiochemists, engineers, radiopharmacists, immunologists, structural biologists, health physicists, coordination chemists, etc. Since radionuclide production is the first step of this chain, it requires an interface between all these disciplines for the radionuclide in question to reach the clinic.

**Figure 1:**
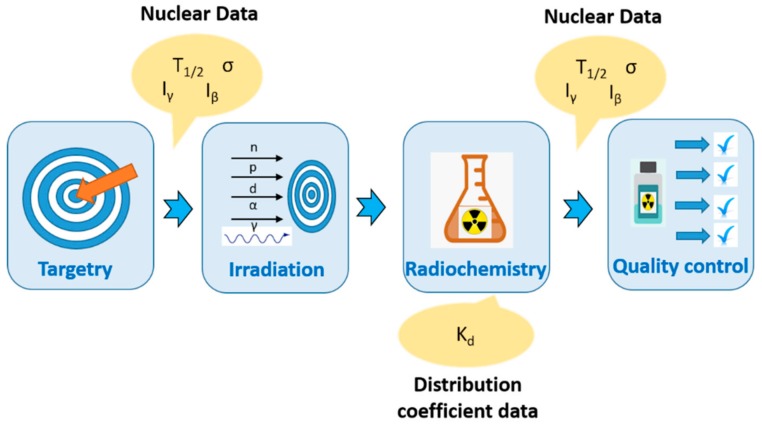
Stepwise radionuclide production of medical radionuclides (T_1/2_: half-life, σ: cross-section, I_γ_ and I_β_ branching ratios).

The production of novel radionuclides for medical applications essentially consists of four successive steps: targetry and irradiation of targets (including cross-section measurements and production rates), radiochemistry (chemical separation) and quality control (Figure 1). Production of sufficient amounts and high quality of novel radionuclides requires systematic research in each of these steps. The selection of radionuclide candidates requires careful considerations based on several factors:

1) Radiation characteristics such as the type(s) of decay emission(s), the energy of the emission(s), LET, physical half-life. If a gamma-ray photon is emitted in the decay process, its energy and branching ratio should be in the diagnostically useful range (dose received by the patients should be minimized, photon energy should be mostly less than 400 keV [38]). The physical half-life of the therapeutic radionuclides is extremely important for minimizing decay loss and provides logistical advantages for the shipment. Most of the novel radionuclides have relatively long half-lives, however, in some cases, due to their short half-life, irradiation facilities should be on-site.
2) The cost of the target material and stable long-term supply.
3) Availability of the facility (production capability and logistics).
4) A reasonable production cost.
5) The chemical separation method should be as simple as possible, for remote handling and to produce no-carrier-added (n.c.a.) products.

### 2.1. Targetry

Targetry is the first step for radionuclide production. Currently, the sources of the radionuclides produced are nuclear research reactors and cyclotron facilities using protons, deuterons and alpha particles. The use of electron linear accelerators are on the rise. Radionuclide generators are also commonly used; however, they still need a reactor or cyclotron source to produce the parent radionuclide. The choice of target material for the production is key, as the irradiation parameters, as well as the subsequent devised chemical separation, are affected.

#### 2.1.1. Target Preparation for Cyclotrons

##### Solid Targets

Most novel radiometals are produced using solid target systems. Methods for solid target manufacture are usually classified as mechanical, physical and chemical. Several different methods such as sintering, rolling, melting, sedimentation, mechanical pressing, electrodeposition, magnetron sputtering, etc. are used for the cyclotron solid target preparation [39,40]. The use of mechanical methods (rolling and pressing) is most common, which consists of a mechanical reshaping of solid or powder materials to produce self-supporting targets. The following criteria are important for the design of the solid target to be irradiated:

1) Selection of the target and backing material: target and backing material are chosen based on high stability and high melting point. In addition, target material should have a good heat conduction capability at the interface of the target and backing material to have efficient heat transport. Targets must be mechanically stable and resistant to radiation damage. The chemical form of the target material should be compatible with the post-irradiation processing, while backing material (if used) should be chemically inert in the respective dissolution process. Target material must be as chemically and isotopically pure as reasonably possible. Impurities can enhance the production of undesired side products, which could impact the radionuclidic purity and subsequent labeling process. In addition, target uniformity, homogeneity and contaminants strongly depend on the target preparation method.

2) Target thickness: target thickness is another important parameter for radionuclide production. It is possible to minimize the side products, depending on the chosen energy range and the thickness of the target. Optimal target thickness can be evaluated using SRIM software by taking the irradiation angle of the beam into consideration [41].

3) Efficient cooling: when targets are irradiated, it generates heat. Cooling systems should be such that the irradiated target retains its integrity as much as possible. Especially for target materials with a low melting point, the surface temperature of the target can be critical.

##### Liquid Targets

Liquid target systems, while popular for the production of ^18^F using enriched water, are also used for the production of radiometals such as ^68^Ga, ^64^Cu, ^61^Cu, ^89^Zr, ^44^Sc, ^86^Y,^63^Zn and ^94m^Tc [42,43]. They can shorten the pre- and post-irradiation target preparation steps and simplify the target transfer after irradiation [42]. In addition, liquid targets have the advantage of producing radiometals with existing medical cyclotrons without investment for a solid-target station [44]. Liquid targets should be prepared such that saturation point isn’t reached such that precipitation occurs.

#### 2.1.2. Target Preparation for Reactors

When using reactors to irradiate target material, they are encapsulated in high purity quartz ampoules in powder form. Before irradiation, the quartz ampoule should be carefully cleaned to avoid the activation of potential impurities. The target material is often initially dissolved, placed into the ampoule and carefully evaporated to dryness. The ampoule is sealed and placed in an aluminum capsule, which is also sealed by electron-beam welding. The chemical purity of the product strongly depends on the opening of the quartz ampoule [45]. The low cross-section data of a radionuclide imposes the use of as large amounts of target material as reasonably possible. Small amounts of target material can also be compensated by high neutron beam flux of the reactor, however.

### 2.2. Production

Production yield depends on the beam flux, energy, irradiation time and the number of target nuclei, respectively. To determine the most efficient production routes and to minimize the number and amount of side products, nuclear reaction cross-section data of the target material have crucial importance. It should be pointed out that the inherent success of any production strategy requires precise nuclear data [46,47,48]. Between 2012 and 2016, extensive nuclear data studies were carried within a research project, coordinated by the International Atomic Energy Agency (IAEA) consisting of compilation, evaluation, and recommendation of cross-section data for the production of medical radionuclides [49,50]. As a result, considerable extensions and valuable improvements have been made to the IAEA-NDS recommended cross-section database for the production of PET and gamma-emitting radionuclides.

It is important to investigate all the possible production routes and then to choose the most proper reaction and energy range. However, even after optimization of all the parameters, the experimental production yields are lower than the theoretical thick target yields due to several factors such as loss of beam particles, radiolysis, variation of the beam intensity, density reduction and/or evaporation in the beam path, etc. [51,52].

#### 2.2.1. Cyclotrons

Neutron deficient radionuclides are produced using a cyclotron and they decay by electron capture (EC) or β^+^-emission. The majority of radionuclides produced using cyclotrons utilize the “Target Material (p,xn)Desired Radionuclide” nuclear reaction. Cyclotrons are classified into three categories, namely, small medical cyclotrons (< 20 MeV), intermediate-energy cyclotrons (20–35 MeV) and high-energy cyclotrons (>35 MeV). According to the report of Goethals and Zimmermann 1050 small medical cyclotrons, 100 intermediate-energy cyclotrons and 50 high-energy cyclotrons were reported worldwide in 2015 [53].

Depending on the type of cyclotron being used, the proton energy can be degraded to lower energies with selected materials to be able to investigate different production routes [54]. In some facilities, deuterons and helium also have been used as a projectile. However, their availabilities are limited compared to proton accelerators.

#### 2.2.2. Nuclear Reactors

Neutron-rich radionuclides are produced by nuclear reactors and they decay by β^—^emission. The majority of radionuclides produced using nuclear reactors utilize the “Target Material (n,γ)Desired Radionuclide” nuclear reaction. Nuclear research reactors are also classified into three categories, namely, low neutron flux (<10^13^ cm^−2^s^−1^), medium neutron flux (2–8 × 10^13^ cm^−2^s^−1^) and high neutron flux (>10^14^ cm^−2^s^−1^) reactors. Several imaging or therapeutic medical radionuclides (^99^Mo/^99m^Tc, ^90^Y, ^131^I, ^153^Sm, ^166^Ho, ^177^Lu, ^161^Tb, etc.) are currently produced using nuclear reactors.

According to the International Atomic Energy Agency (IAEA) Research Reactor Database, 97 research reactors are reported to be in use towards radionuclide production [55]. Krijger et al. reviewed the status and necessity of nuclear reactors for targeted radionuclide therapy [56]. The availability of the high thermal neutron flux (>10^14^ cm^−2^s^−1^) nuclear research reactors is especially important for the production of medical radionuclides with high specific activities. Most of the nuclear research reactors currently in service for this purpose, however, were built in the 1960s and they require longer and more frequent shut-down periods for maintenance to continue operation with minimum failure, which limits their radionuclide production capability considerably. Considering the expanding growth of the nuclear medicine market, it is foreseen that the capacity of the remaining nuclear research reactors will not fulfill the demand for therapeutic radionuclides in the near future.

#### 2.2.3. Alternative Production Routes

In some cases, it is difficult to produce carrier-free or radionuclidically pure products with conventional reactor-based or accelerator-driven production routes. In this case, the use of mass separators to produce carrier-free radionuclides for nuclear medicine is becoming more and more attractive. However, this method requires large-scale mass separation facilities which are available for this purpose in few places worldwide: MEDICIS at CERN [57,58,59,60,61], ISAC at TRIUMF, Canada [62], FRIB (under construction in Michigan, U.S.A.), ISOL at MYRRHA (under construction in Mol, Belgium) [63] and SPES-INFN (under construction in Legnaro, Italy). At these facilities, radionuclides are produced via high-energy proton induced reactions combined with an online mass separator, known as Isotope Separation On-Line (ISOL). Alternatively, it has been considered to use external proton or neutron-irradiated targets to perform offline mass separation to enhance the possibilities of producing promising novel radionuclides with the mass separation technique [64]. The mass separation technique is based on the ionization processes such as surface, plasma or laser ionization, however, these methods are not element selective and could cause isobaric contamination in the resultant material. As a result, radiochemical separation processes must still be developed to use mass-separated radionuclides for nuclear medicine applications. Other than cyclotron and nuclear reactor production routes, there are also much less common alternative production routes such as the use of electron linear accelerators [65,66], Van der Graaf accelerators [67] or lasers [68,69,70] to produce radionuclides for nuclear medicine applications.

### 2.3. Radiochemistry

Radiochemistry is used for two purposes, to isolate the desired radionuclide in n.c.a. form, by means of chemical separation, and to recover the enriched target material for reuse.

Different chemical separation techniques have been considered to obtain n.c.a. radionuclides, such as ion-exchange chromatography, extraction chromatography, solvent extraction, precipitation, and electrochemical methods. The selection of the chemical separation process depends on several parameters:

1) Adaptation of the separation using a remote/semi-automated operation system;
2) Separation processing time;
3) Product yield as a result of the separation process;
4) Recovery of enriched target material for recycling;
5) The chemical form of the final product;
6) Acidity and the volume of the final product;
7) Robustness and reproducibility of the process.

Ion exchange and extraction chromatography are the most convenient methods for the remote/semi-automated separation and purification operations. In this case, distribution of an element between the solution and ion exchange resin, which is defined as distribution coefficient data, has a great value to define the separation process (Figure 1).

The processing of the irradiated target material requires hot cells in order to decrease the radiation exposure of the operators. Removal of the irradiated target from the target station is performed remotely and is transferred to a reception hot cell. It is very important to eliminate the risk of contamination during target removal and in the processing thereof [71].

### 2.4. Quality Control of the Radionuclide for Preclinical Studies

After radiochemical separation, the quality of the final product requires assessment. This is a vital step for the development of novel radionuclides towards nuclear medicine applications. As part of the quality control process, radiochemical purity, specific activity, radionuclidic purity, and chemical purity measurements should be performed and radiolabeling processes should be established.

Radiochemical purity is defined as the fraction of the total radioactivity of the radionuclide concerned in the sample, which is present as its desired chemical form. It is generally tested by radio-chromatographic methods, such as thin-layer chromatography (TLC) or high-performance liquid chromatography (HPLC).

Specific activity is defined as radioactivity per unit mass of the product [72]. Several factors that could affect the specific activity of the final product include cross-sections, target impurities, secondary nuclear reactions, target burnup, and post-irradiation processing periods [20].

Accurate activity measurement is crucial for activity dosing and absolute image quantification. According to the international guidelines, the dose of radiopharmaceuticals should be measured with a minimum accuracy of 5 to 10% [73,74,75]. Radionuclide calibrators consisting of ionization chambers are used to measure the product activity. They can be used over a wide range of activities from hundreds of kBq up to tens of GBq. Typically, calibration factors are determined by direct measurement of the samples with a primary standard. Moreover, precise nuclear data information such as decay emission(s), the energy of the emission(s) and half-life are important to perform precise activity measurement.

Activity measurement also requires regular quality control tests (accuracy, geometry, linearity, and constancy) to avoid wrong activity measurements. Geometry effects (same reading for the same amount of activity irrespective of the volume or the vial of the sample) can dramatically affect the activity measurements, particularly for low-energy X-ray or gamma photons. It should be, therefore, assessed for the vials used to collect the product, as well as those used for quality control and in GMP [76,77].

Radionuclidic purity is defined as the ratio of the radioactivity of the desired radionuclide to the total radioactivity content of the sample. Gamma spectroscopy is the main instrument used for the determination of the radionuclidic purity. Efficiency calibration of the detector system must be properly performed with calibrated standard gamma sources. Measurements should be repeated after an appropriate decay time to determine long-lived contaminants.

Chemical purity is controlled by the determination of the impurities in the final product. It has explicit importance and has a direct effect on the radiolabeling of the final product to a target molecule. The macrocyclic chelators are not selective for the radiometal of interest and they can coordinate with other (nonradioactive or “cold”) metal ions and depends on the specific activity of the final product, where even ppb level impurities could affect the labeling efficiency [78,79,80]. The shelf life of the product also strongly depends on the chemical purity and specific activity. Other than the initial chemical impurities, the effect of decay products on labeling efficiency should also be investigated. Different analytical techniques such as ICP-OES, ICP-MS, ICP-AES etc. could be used to determine the chemical purity of the final product.

Radiolabeling efficiency is defined as the ability of a chelator to form its radionuclide complex. The final product is mixed with a chelator-(peptide/antibody) and pH is adjusted with a suitable buffer solution. The resultant solution is then heated for a certain time and temperature, based on the radiolabeling kinetics of the chelator used. Several reaction parameters such as the concentration of peptide or antibody, buffer conditions, pH, reaction temperature and reaction time can affect the labeling efficiency. In particular, pH plays a critical role in radiolabeling, particularly on the rate of complex formation. In addition, radical scavengers such as gentisic acid and ascorbic acid are used to increase the stability of the labeled compound. Ethanol has also been used to improve the radiolabeling efficacy and to prevent radiolysis [81].

### 2.5. Automation of the Labeling Procedure Towards GMP Production

Radiopharmaceutical products for clinical applications must follow the universal requirements for the drug products. Drug products must for the sake of patient safety be of good quality and have a favorable risk-benefit balance when overall efficacy and safety are assessed. The European Pharmacopoeia (EP) and likewise the United States Pharmacopoeia (USP) and the Japanese Pharmacopoeia (JP) set the minimum quality standards for drug products in general and also for radiopharmaceuticals. When developing new radionuclides intended to be used in new drug products, these monographs should be considered amongst others: dosage form - EP 0520 “Parenteral Preparations”; drug product classes - EP 0125 “Radiopharmaceutical Preparations” and for specific guidance in quality control of such products EP 2.2.66. “Detection and Measurement of Radioactivity”. Recently the European Directorate for the Quality of Medicines & HealthCare (EDQM) published the “Guide for the elaboration of monographs on Radiopharmaceutical Preparations” [82], which is a useful resource for setting quality parameters and specifications for new radiopharmaceuticals.

Apart from the pharmaceutical regulations, it is of key importance to also consult the national radioprotection legislation. The endeavor to translate new radionuclides into the clinic is certainly a procedure that involves many steps. When planning for the first-in-man clinical trial, the quality of labeling solution, as well as overall product quality, should be assessed against EP quality standards to guarantee a good quality product that reproducibly meets all the predefined specifications. When evolving towards later stages of clinical studies and especially towards commercialization of a drug product, full GMP will apply as published by the European Commission’s EudraLex volume 4 of “The rules governing medicinal products in the European Union” [83].

Once a novel radionuclides such as ^161^Tb or ^44^Sc is introduced into a first clinical trial all changes in the manufacture of radionuclide and drug products with potential impact on the quality of the product should go through a formal internal assessment before such changes are applied. Such changes may be an improvement of the targetry, change of the supplier of the target material, changes of solvents or their quality or change of the drug product vial. The assessment should cover aspects like re-validation of processes and quality control (QC) methods, adaptation of specifications and overall impact on the quality of the drug product. Product specifications include [83]:

1) appearance;
2) pH;
3) radiochemical purity/yield;
4) radionuclide identity/radionuclidic purity;
5) radioactivity concentration;
6) tracer identity/quantification;
7) molar activity;
8) chemical purity;
9) residual solvents;
10) bacterial endotoxins (BET);
11) sterility;
12) product stability;

To verify compliance with the specifications, controls must be conducted by suitable and documented procedures for starting materials, including the radionuclide precursors, and final products. Moreover, controls have to be operated also on equipment that has been qualified for the tests to be applied to the actual radiopharmaceutical or radionuclide produced [83]. The validation characteristics such as accuracy, precision, specificity, detection limit, quantification limit, linearity, and range should be considered [82]. QC is that part of GMP which is concerned with sampling, the mentioned specifications of each quality parameter (see above) and with the organization, documentation, and release procedures which ensure that the necessary and relevant tests have been actually and successfully carried out [84]. All the test methods require validation and records to demonstrate that all the steps (sampling, inspecting, testing procedures) were properly carried out and assessed against the defined specifications. Pharmaceuticals are not released to the clinics for the patient use until their quality has been shown to fulfill the required quality. However, in the case of radiopharmaceuticals QC with ultra-short-lived radioisotopes (i.e., positron-emitting radioisotopes ^11^C, T_1/2_ = 20 min; ^18^F, T_1/2_ = 110 min, etc.), it is necessary to administer the product before all the tests have been completed. As described earlier, a very important requirement is that records of all the controls should be reported [82].

Automated modules are regularly used to ensure consistency of labeling the radionuclide to the target to produce the radiopharmaceutical. The so-called GMP module is utilized for the pharmaceutical labeling of a limited number of radionuclides for clinical diagnosis and therapy, namely, ^18^F, ^11^C, ^15^O, ^13^N, ^68^Ga, ^99m^Tc, ^90^Y and ^177^Lu [85,86,87,88].

Stringent regulatory demands make fully-automated approaches necessary for the synthesis of radiopharmaceuticals to be used in clinics. In addition, an automated system can minimize human intervention and guarantee better control of sterility and pyrogenicity of radiopharmaceuticals [89]. A current trend in the development of automated systems is moving from the fixed tubing system, that have characterized a large variety of synthesis modules over the years, to a “disposable sterile cassette”-based system [90]. The disposable cassette systems are intended for single-use and are designed for one or more specific syntheses. ^68^Ga/^177^Lu/^90^Y/^111^In DOTA-peptides have been synthesized with high purity, high radiochemical yield and acceptable synthesis time using such a system [91].

The development of an automated procedure for therapeutic radiolabeling with novel radionuclides is a considerable challenge and several factors have to be taken into consideration. Firstly, the final product has to be purified from possible free activity (radionuclide that is not associated with the conjugate molecule), which is performed using cartridge-based methods. Furthermore, to achieve high labeling efficiencies of conjugates, the concentrations of the reagents used must be high and, therefore, low volumes are used. These small volumes of reagents require transfer through the system without or minimal loss of solution. Another factor to keep in mind is the number of reagents that a radiolabeling procedure typically requires [92]:

1) the conjugate molecule;
2) the radionuclide;
3) a buffer to maintain pH;
4) a scavenging agent (such as ascorbic acid) to prevent radiolysis;
5) a quenching agent (such as EDTA) to complex any radionuclide that is not associated with the conjugate molecule;
6) diluent to formulate the preparation for administration to patients.

It is also important to prevent contamination of the product with metallic impurities, which may compete with the radionuclide for the limited number of chelating sites on the conjugate molecule [79]. This implies that metallic transfer systems (such as needles) ought to be avoided where transferring the acidic radionuclide solutions (pH 1–2). In parallel to the development of the automated synthesis procedure, the appropriate quality controls have to be established and validated. In the following section, the current status and the recent developments for the production of ^44^Sc and ^161^Tb will be presented in comparison with ^68^Ga and ^177^Lu, respectively.

## 3. The Diagnostic Radionuclides ^68^Ga and ^44^Sc

### 3.1. Targetry/Production

Table 2 shows the comparison of ^68^Ga and ^44^Sc and decay characteristics and production routes. To date, several different target materials are considered for ^68^Ga, ^44^Sc production (Table 3).

#### 3.1.1. ^68.^ Ga Generator Production

Commercially available ^68^Ge/^68^Ga generators are produced via ^69^Ga(p,2n)^68^Ge reaction by irradiating ^nat^Ga with 23 MeV proton energy [89]. The ^68^Ge (T_1/2_ = 270.95 d) produced is separated from the Ga target material [105,106] and the final product loaded onto an inorganic substrate (SnO_2_ or TiO_2_) which is shielded with Pb. ^68^Ge decays by electron capture to ^68^Ga (T_1/2_ = 67.71 min) and the daughter is subsequently eluted from the resin using dilute HCl (0.1 -0.6 M HCl) which is introduced to a GMP module for radiolabeling. Alternatively, ^68^Ge can be produced with high energy protons utilizing the ^71^Ga(p, 4n)^68^Ge reaction or with an alpha beam via the ^66^Zn(α, 2n)^68^Ge nuclear reaction [107].

#### 3.1.2. ^68.^ Ga Cyclotron Production

*Solid targets:* production of ^68^Ga is also possible by using enriched ^68^Zn or ^nat^Zn targets using the ^68^Zn(p,n)^68^Ga nuclear reaction with a medical cyclotron at ~12 MeV (to ensure no ^67^Ga production via the (p,2n) reaction at >12 MeV) [101]. However, even using enriched ^68^Zn targets, the co-production of ^66^Ga (T_1/2_: 9.49 h) and ^67^Ga (T_1/2_: 3.26 d) cannot be avoided. Previously, electroplating of ^68^Zn on different backing materials such as copper, silver and platinum were reported [101,102,103]. Using natural copper and silver as a backing material would lead to the production of long-lived ^65^Zn (T_1/2_ = 244 d) and ^109^Cd (T_1/2_ = 453 d) via the ^65^Cu(p,n)^65^Zn and ^109^Ag(p,n)^109^Cd nuclear reactions, respectively. Platinum backing has a lower thermal conductivity than copper and silver, however, it is considerably more expensive. ^68^Zn foil targets can also be used directly for ^68^Ga production [98], nevertheless, commercial availability of enriched Zn foil is limited. In a recent study, enriched ^68^Zn pressed targets were also used for the large-scale production (up to 140 GBq) of ^68^Ga [108]. Effective molar activity was reported as 77.4 ±5.0 GBq/μmol with 89% recovery yield.

*Liquid targets:* alternatively, the production of ^68^Ga by using liquid zinc chloride and zinc nitrate targets with medical cyclotrons at ~ 13-14 MeV have been reported [42,93,95,96,97]. Zinc chloride solution can produce high target pressure during irradiation by increasing gas evolution and it is highly corrosive [97]. On the other hand, nitrate ions decrease the gas evolution by increasing the hydroxyl radical formation in the solution, which makes it a better option compared to zinc chloride targets. Alveset al. reported that up to 24 TBq/µg specific activities of ^68^Ga can be reached by using liquid ^68^Zn(NO_3_)_2_ targets [42].

Cyclotron production of ^68^Ga has the advantage of extremely high activities of ^68^Ga over eluting product from a generator.^44.^ Sc generator production

#### 3.1.3. ^44^Sc Generator Production

^44^Sc generators are produced via the ^45^Sc(p,2n)^44^Ti nuclear reaction.^44^Ti (T_1/2_ = 60.6 a) decays by electron capture to ^44^Sc, which subsequently decays to stable ^44^Ca. The advantage of this method is the target material ^45^Sc, which is the only naturally occurring stable isotope of scandium; therefore, it does not require expensive enriched target material for irradiations. This reaction has a low production rate and requires high proton flux and a long irradiation time for the production of ^44^Ti with high radioactivity yields [109,110]. Production of 185 MBq ^44^Ti was reported with an internal proton beam of 28.5 MeV and 60 and 500 µA external and internal proton fluxes [111].

Alternatively, ^44^Ti can be produced with high energy protons using vanadium or iron targets thanks to spallation reactions (^51^V(p,2p6n)^44^Ti and ^54^Fe(p,^11^B)^44^Ti). This process requires a complex radiochemistry for the purification of ^44^Ti from proton irradiated target material, however [112,113,114].

A 370 MBq (10 mCi) generator should be able to be eluted every 4 h to provide 148-185 MBq (4–5 mCi)—the equivalent of a single patient dose [115]. In addition, the lifetime of ^44^Ti/^44^Sc generator is longer than the one of ^68^Ge/^68^Ga due to the long half-life of ^44^Ti. Nevertheless, the co-production of longer-lived radionuclidic impurities such as ^48^Sc (T_1/2_ = 43.7 h) and ^44m^Sc (T_1/2_ = 58.5 h) would cause an additional dose burden to the patients.

Generator-produced ^44^Sc has been used for preclinical investigations [116] and the first ^44^Ti/^44^Sc generator used for such purpose achieves elution of 180 MBq ^44^Sc with 97% yield and the breakthrough of ^44^Ti was reported as 90 Bq. [109]. In 2012 the first clinical application of [^44^Sc]Sc-PSMA-617, utilized with generator-derived ^44^Sc, occurred for PET imaging of metastasized castrate-resistant prostate cancer, as a proof-of-principle study [35]. The qualities of the images obtained with [^44^Sc]Sc-PSMA-617 were comparable with those of [^68^Ga]Ga-PSMA-11.

#### 3.1.4. ^44.^ Sc Cyclotron Production

*Solid targets:*^44^Sc production via proton irradiation of natural metallic calcium targets (^nat^Ca(p,n)^44^Sc) was investigated by Severin et al. and Valdovinos et al. [117,118]. Severin et al. reported a ^44^Sc production yield of more than 650 MBq with a 70% yield. This process does not need target recycling, however, radionuclidic purity of the final product (95%) is not regarded as sufficient for clinical application. Other than the relatively low production yield, the use of natural calcium leads to the co-production of long-lived radionuclides, namely, ^44m^Sc, ^47^Sc (T_1/2_ = 80.4 h) and ^48^Sc, which can lead to additional dose burden to patients.

The use of enriched ^44^Ca targets was considered a better option for ^44^Sc production (^44^Ca(p,n)^44^Sc), available commercially in carbonate form [99,100]. One of the drawbacks of using CaCO_3_ as a target material is the release of CO_2_ during irradiation, which could cause the distortion of the target capsule. To avoid this from occurring, enriched ^44^CaCO_3_ was annealed at 900^o^C to convert it to ^44^CaO, which was then used as the target material [104].

Alternatively, Duchemin et al. and Alliot et al. investigated the production cross-section of deuteron-irradiated ^44^Ca targets (CaCO_3_) [119,120]. Alliot et al. demonstrated that ^44^Sc production with deuterons leads to higher cross-section and higher radionuclidic purity in comparison to that of proton irradiation [120]. Production of Sc radionuclides via α-particle irradiation was also investigated and it was demonstrated that the ratio of ^44m^Sc/^44^Sc is five and twenty times higher in comparison with deuteron and proton-induced reactions, respectively [121].

*Liquid targets:* Production of ^44^Sc with liquid targets (natural Ca(NO_3_)_2_ in ultrapure water) was investigated utilizing the ^44^Ca(p,n)^44^Sc nuclear reaction. [94]. The production yields reported (28 ± 1 MBq) were much lower than those reported using solid targets.

### 3.2. Radiochemistry

#### 3.2.1. ^68.^ Ge/^68^Ga Generator Production

The success of radionuclide generators depends on the selection of the parent/daughter pair and the radiochemical separation procedures. Dash et al. summarized the selection criteria for the radiochemical separation method for radionuclide generators [122]. Radiochemical separation method should be fast, reproducible and provide the daughter radionuclide in high radionuclidic, radiochemical and chemical purity.

The ^68^Ge product is subsequently loaded onto a shielded column containing, predominantly, SnO_2_ or TiO_2_ as matrix – to create what is known as a generator system. The ^68^Ge is left on the column matrix to decay to its daughter, ^68^Ga, which is then eluted (or “milked”) as ^68^Ga^3+^ with 0.1 M HCl. The breakthough of ^68^Ge from the TiO_2_ column is reported as ~ 10^−3^ % of the eluted ^68^Ga activity [123]. Due to the presence of metallic impurities such as Fe(II), Mn(II) and Zn(II), post processing of the eluate (^68^GaCl_3_) is required for high labeling yield and high specific activity [124].

#### 3.2.2. ^68.^ Ga Cyclotron Production

Target material should be processed to remove the bulk zinc and other metal impurities prior to radiolabeling process of ^68^Ga. To date, several different separation techniques such as ion exchange chromatography, solvent extraction [125,126], thermal diffusion [127] and precipitation [128] techniques are used for the separation of Zn from Ga. Two-step column separation systems have been considered for the ion exchange method. The first column (AG50W-X8 cation exchange resin or Hydroxamate) has been used to separate macro quantities of Zn from ^68^Ga, while the second column (AG1-X8 anion exchange resin, DGA or UTEVA extraction resins) is used to further reduce any traces of metallic impurities, while also decreasing the acidity and the volume of the final product [96,97,103]. Comparison of the chemical purities in generator- and cyclotron produced ^68^Ga solutions showed that zinc content was significantly lower for cyclotron-produced ^68^Ga [103].

##### Recycling of ^68^Zn

The price of commercially available enriched ^68^Zn is relatively low. As a result, the facilities utilizing this method do not currently recycle the target material [103].

#### 3.2.3. ^44.^ Sc Generator Production

Due to the long physical half-life of ^44^Ti, the long-term stability of the generator is essential. The separation process should provide high ^44^Sc elution and zero-to-low ^44^Ti breakthrough. The radiochemistry for the generator system consists of a column containing AG1-X8 anion exchange resin. ^44^Ti is dissolved in 0.1 M H_2_C_2_O_4_ and loaded onto the column. Once secular equilibrium was reached ^44^Sc could be eluted with 20 mL of 0.005 M H_2_C_2_O_4_/0.07 M HCl solution, with >97% elution yield (180 MBq) and low ^44^Ti breakthrough (5 × 10^−5^ % or 90 Bq) [109]. It was discovered, however, that the eluate was too dilute and acidic for direct labeling. As a result, a post elution processing step was developed, with purified ^44^Sc eluted with 3 mL 0.25 M ammonium acetate buffer (pH = 4.0) [110].

A gas-phase separation method for the separation of scandium from titanium foil has also been proposed, via thermal release in vacuum [129]. It was shown that by heating around 1200 ^°^C for one hour, almost all the scandium is released from titanium matrix.

#### 3.2.4. ^44.^ Sc Cyclotron Production

Cyclotron production of ^44^Sc requires an efficient ^44^Sc/Ca separation procedure. Severin et al. reported separation of Sc by means of precipitation. After irradiation, natural calcium was dissolved in HCl and the Sc separated from Ca by its precipitation as Sc(OH)_3_ at neutral pH [117]. Different ion exchangers such as UTEVA, Chelex 100 and DGA resins are also commonly used to separate Sc from Ca [99,100,118,120], however, to date, DGA resin is the most commonly-used system for the separation of Sc [99,120]. It ensures elution of Ca in more concentrated HCl, while Sc can be eluted further with 0.1 M HCl. Van der Meulen et al. reported a fast and efficient Sc/Ca separation method with 98% efficiency [99]. After dissolving CaCO_3_ in 3.0 M HCl, ^44^Sc is separated from the target material using DGA resin, after which it is concentrated using SCX resin. Production of up to 2 GBq ^44^Sc was reported with high radionuclidic purity.

##### Recycling of ^44^Ca

The natural abundance of ^44^Ca (2.086%) is much lower than ^68^Zn (19.024%), therefore, recycling of the enriched ^44^Ca is important to decrease the cost of production. A simple recycling procedure was reported: firstly, calcium is precipitated as Ca-oxalate, after which it is converted to carbonate by slowly heating to 500 ^º^C [99].

### 3.3. Labeling Efficiency

#### 3.3.1. ^68.^ Ga

The end product for both cyclotron-produced and generator-eluted ^68^Ga is in chloride form. The main issue in the production process of ^68^Ga radiopharmaceuticals is the elution from the ^68^Ge/^68^Ga generator. While this is a popular means of obtaining the PET radiometal, there are some drawbacks using the ^68^Ga eluate for radiolabeling. The most pressing issues to be addressed are the large volume of the generator eluate (approximately 5 – 10 mL), the HCl concentration (0.1 – 1.0 M in metal oxide-based generators) and the presence of measurable activity of the long-lived parent radionuclide ^68^Ge (i.e., ^68^Ge breakthrough). Other metallic impurities generated from the generator columns (Ti^4+^), from the decay of ^68^Ga (Zn^2+^), or introduced as a contaminant in the process (Fe^3+^) may adversely affect the ^68^Ga labeling reaction, as well as the molar activity of the labeled product. As a result, the post-processing of the product is necessary, as mentioned previously. The eluate is loaded onto a cation exchange resin and the desired product separated from the impurities above [130]. The use of automated systems ensures high yields and safe preparation of ^68^Ga-labeled radiopharmaceuticals for routine application [90].

Schultz et al., developed a method for radiolabeling of DOTA-like conjugated peptides with generator-produced ^68^Ga, using NaCl-based cationic elution method. This method has an advantage of reducing preparation time, thanks to the absence of organic solvent in the eluent [87]. Although DOTA-peptides are the most widely used ones, it was shown that TRAP chelators have a better binding ability for ^68^Ga compared to DOTA and NOTA-peptides [131]. Similarly, under milder conditions, the synthesis of ^68^Ga(THP-TATE) is significantly faster compared to the ^68^Ga(DOTATATE) [132]. In a recent study, Sinnes et al., have shown that ^68^Ga(DATA-TOC) can be prepared under milder conditions than the ^68^Ga(DOTA-TOC), which has a practical advantage for instant kit-type labeling without effecting the efficacy [133].

#### 3.3.2. ^44.^ Sc

The optimum conditions for the labeling of ^44^Sc with DOTATOC, DOTA, and DOTATATE were reported in refs [134,135]. It was also demonstrated that ^44^Sc can be used for the labeling of biomolecules with both DOTA and NODAGA chelators [136]. It was reported elsewhere that DOTA is the most suitable ligand among a series of macrocyclic ligands for binding scandium radionuclides [137]. The stability study in the presence of rat serum demonstrated that ^44m/44^Sc-DOTATATE is stable over a period of up to 25 h [135]. Similar results were obtained by van der Meulen et al. for ^44^Sc-DOTANOC, which showed stability over 24 h. Moreover, an improved scavenging effect was observed with gentisic acid compared to ascorbic acid [99].

pH plays a critical role in radiolabeling of Sc, particularly on the rate of complex formation. Scandium is known to be a lanthanide-like element due to their similar chemistry, however, it forms different hydroxide complexes at the same pH in comparison to lanthanides. At pH 4.5 lanthanides are present only as Ln^3+^ [138], while Sc is present in different chemical speciations such as Sc^3+^, Sc(OH)^2+^, Sc(OH)_2_^+^ and insoluble Sc(OH)_3_ [139,140]. Taking this into consideration, optimal pH for Sc labeling should be lower than 4.5.

### 3.4. Production for the Clinical Applications

#### 3.4.1. ^68.^ Ga

The first pharmaceutical-grade ^68^Ga generator (Galli Ad) received a Marketing Authorization as a drug product by the EMA in 2014 [141]. Details of the commercially available ^68^Ge/^68^Ga generators are reported in [141]. The existing [^68^Ga]GaCl_3_ European Pharmacopeia monograph is based on the commercially available ^68^Ge/^68^Ga generators. Nowadays, many ^68^Ga-labeled tracers are used in clinical trials and NETSPOT and SomaKit TOC™ have already been approved by the FDA and EMA, respectively [142].

Several approaches for processing generator-derived ^68^Ga eluates have been recently described, each of them with a markedly different impact in terms of technology and engineering towards the development of automated systems for ^68^Ga radiolabeling [90]. The product is labeled to the conjugate in question, in a similar manner to that described previously, and the product obtained then purified from any unlabeled ^68^Ga using a C18 cartridge. The radiopharmaceutical is eluted from the C18 cartridge using ethanol or an ethanol/water mixture and eventually diluted with 0.9% saline as the final product. To ensure sterility, the final product is eluted through a 0.22 μm sterilization filter. Instant thin layer chromatography (ITLC) as well as HPLC are performed as a quality control to ensure the radiochemical purity (RCP) of the product is above 95% and the fraction of free ^68^Ga^+^ or its colloids is less than 2%, to allow the release of the product for clinical administration. Germanium breakthrough needs to be assessed with the quantity limited to a maximum of 0.001% [143,144,145].

As it was mentioned in Section 2.4, the precise activity measurements are essential for dosing and absolute image quantification. In a recent study, accuracy of four different types of radionuclide calibrators was investigated for ^68^Ga by using well-calibrated gamma spectrometry. The results were surprising in that all the radionuclide calibrator systems showed a systematic error in the range of 10–25% [76,146]. It was reported that the deviations were mainly due to the incorrect factory-shipped dose calibrator setting.

At the time of writing, a specification exists only for the generator-produced ^68^Ga in the European Pharmacopoeia monographs (Table 4), however, a new monograph of the European Pharmacopeia is in preparation for accelerator-produced ^68^Ga solution [75]. Specifications for generator or cyclotron produced ^68^Ga are similar except for the radionuclidic purity, which is proposed to be >98% (<2% ^66^Ga and ^67^Ga) for cyclotron-produced ^68^Ga (Table 5).

#### 3.4.2. ^44.^ Sc

There is no monograph in the European or another pharmacopeia available for the preparation of ^44^Sc or ^44^Sc-radiopharmaceuticals. To date, the quality control was performed based on the monograph for [^68^Ga]Ga-DOTATOC of the European Pharmacopeia. As Sc is chemically more similar to the lanthanides than Ga, the labeling procedure would be potentially similar to that of ^177^Lu. The labeling pH should be taken into consideration as was pointed out in the previous section. Thanks to the promising preclinical studies with ^44^Sc, proof-of-principle first-in-man studies were performed with [^44^Sc]Sc-PSMA-617 and ^44^Sc-DOTATOC [35,149], demonstrating that PET/CT imaging can be performed several hours post-injection (19 and 24 h) thanks to the longer half-life of ^44^Sc.

## 4. Beta-Emitting Radionuclides: ^161^Tb and ^177^Lu

### 4.1. Targetry Production

#### 4.1.1. ^177^Lu

The main methods for producing ^177^Lu with high specific activity are based on neutron irradiation of either enriched ^176^Lu or ^176^Yb in reactors. Lu_2_O_3_ or Yb_2_O_3_ are used as target material, due to their thermal stability (during irradiation) and solubility in diluted mineral acids [10].

##### Direct Method: Production of Carrier-Added (c.a.) ^177^Lu

The direct production of ^177^Lu takes place via the ^176^Lu(n,γ)^177^Lu nuclear reaction. ^176^Lu has a very high thermal neutron capture cross-section (σ: 2090 b), which allows reaching high yields and relatively high specific activity. The co-production of ^177m^Lu (T_1/2_ = 160.4 d), however, is an issue for radiation protection and waste disposal (laboratory waste, hospital wastewater). The irradiated material, Lu_2_O_3_, is easily dissolved in concentrated hydrochloric acid and heated until dryness to convert the oxide to chloride. The resultant chloride residue is dissolved in dilute hydrochloric acid and it is used directly for labeling. In this phase of preparation, contamination due to the impurities in the initial material (target material or quartz ampoule) and during the processing of the irradiated material, which can directly affect the labeling efficiency, should be taken into consideration.

Based on theoretical calculations, no long-lived radionuclidic impurities, except for ^177m^Lu, are expected to be found in the irradiated lutetium. Barkhausen et al. performed gamma spectrometry measurements three weeks after irradiation, to confirm these data and the obtained results showed the presence of only ^169^Yb (T_1/2_: 32.026 d) and ^65^Zn (T_1/2_: 244.26 d) as contaminants. However, at the end of irradiation, their relative activities were very low 1.3 × 10^−4^ and 1.8 × 10^−4^ %, respectively [150].

##### Indirect Method: Production of No-Carrier-Added (n.c.a.) ^177^Lu

The production of n.c.a. ^177^Lu occurs by the irradiation of enriched ^176^Yb via ^176^Yb(n,γ)^177^Yb→^177^Lu nuclear reaction. The production yields are much lower compared to the direct method due to the low thermal neutron cross-section of ^176^Yb (2.5 barn). It requires a well-developed radiochemical separation method to be able to reach high labeling efficiencies and for the recycling of the enriched ^176^Yb. Enriched ^176^Yb oxide contains 2–3% of ^174^Yb, which also leads to the formation of ^175^Yb (T_1/2_ = 4.18 d). This method is much more expensive compared to the direct method, however, it ensures production of ^177^Lu with high radionuclidic purity without the presence of long-lived radioactive impurities, which minimizes the radiation protection and waste disposal issues. In addition, the shelf-life of n.c.a. ^177^Lu product is much longer compared to c.a. ^177^Lu.

#### 4.1.2. ^161.^ Tb

Indirect method: production of no-carrier-added ^161^Tb. The production of n.c.a. ^161^Tb takes place with the irradiation of enriched ^160^Gd via the ^160^Gd(n,γ)^161^Gd→^161^Tb nuclear reaction. ^159^Tb is the only stable isotope of terbium, therefore, a direct production route is not an option to produce ^161^Tb.

A method to produce ^161^Tb towards medical application was first proposed by Lehenberger et al. [151]. Table 6 shows the comparison of ^177^Lu and ^161^Tb decay characteristics and their relative production routes. The ^176^Yb thermal neutron reaction cross-section (2.5 barn) is high compared to that of ^160^Gd (1.5 barn). As a result, longer irradiation times or higher flux are required for the production of the same activities for ^161^Tb. The natural abundance of ^160^Gd (21.86%) is higher than ^176^Yb (12.76%), however, which makes the target material to be irradiated less expensive (Table 7).

Production of ^161^Tb was also investigated via deuteron-induced reactions (^160^Gd(d, x)^161^Tb) over a wide energy range up to 50 MeV, using the stacked-foil technique and high-resolution γ-spectrometry [47]. Nevertheless, it was reported that, even when using ^160^Gd enriched targets, the radionuclidic purity of the final product cannot be high due to the co-production of ^160^Tb.

^161^Tb has not yet been listed on the dangerous goods tables of the ADR (European Agreement concerning the International Carriage of Dangerous Goods by Road) and International Air Transport Agency (IATA) regulations, which are based on IAEA (International Atomic Energy Agency) recommendations. As a result, the generic A2 value (activity limit of radioactive material) according to the “Basic Radionuclide Values for Unknown Radionuclides or Mixtures” of 0.02 TBq has to be applied for ^161^Tb transportation [154]. Based on the current limitation of irradiation conditions (due to the transportation limit), the specific activity of ^161^Tb has yet to be optimized.

### 4.2. Radiochemistry

#### 4.2.1. ^177.^ Lu

Due to their chemical similarity, the separation of neighboring lanthanides is challenging. The effective separation of micro amounts of ^177^Lu product from macro amounts of Yb target material, additionally, needs special attention since high separation factors (>10^5^) are required. The most common method to separate lanthanides is the use of α-HIBA (α-hydroxyisobutryric acid) as eluent in a cation exchanger separation system [155]. To reach high separation factors, α-HIBA concentration, pH of the solution and the parameters for the cation exchanger (column volume, flow rate, particle size, etc.) should be optimized.

Several methods have been proposed in the literature for the separation of ^177^Lu from Yb [156,157,158,159,160,161]. Kuznetsov et al., reviewed the separation of Lu and Yb by reduction of Yb with sodium amalgam and subsequent developments [19]. The radiochemical separation method for the routine production of n.c.a. ^177^Lu on an industrial scale for medical applications is described in [162]. The reported separation method is capable of the separation of ^177^Lu and ^176^Yb in a mass ratio of 1:10^2^ to 1:10^10^. It contains a five cation exchange column system. Firstly, Yb_2_O_3_ is dissolved in mineral acid, then, the solution is loaded to the first column system (macroporous cation exchanger in NH_4_^+^ form). The first two columns are used to separate the macro amounts of Yb from the micro amount of Lu. Then, the desired separation factor for Lu is reached with further separation of Yb performed by the next two columns. The α-HIBA is solution used as a chelating agent to separate Lu and Yb. After elution of Lu from the second column, the macro amounts Yb is eluted with a higher concentration of HIBA and collected for recycling.

The final Lu solution is acidified to dissociate the α-HIBA-Lu complex and it is eluted from the fifth column in 1.0 M to 12.0 M mineral acid. The mineral acid is then evaporated and the product prepared by collection in the desired solution. The final product is suited for radiopharmaceutical use without requiring further purification and/or sterilization. It was reported that with this method, 3.9 TBq of ^177^Lu/mg of lutetium could be obtained and more than 400 MBq ^177^Lu could be labeled per µg peptide or other biomolecules [161].

#### 4.2.2. ^161.^ Tb

A two-column separation system has been applied for the separation of terbium from enriched gadolinium target material [162]. After dissolution of target and the loading of the radionuclides onto a Sykam cation exchange resin column, α-HIBA separation system is used to separate terbium from macro amounts of gadolinium target material.

The α-HIBA-Tb complex must be decomposed before labeling. This compound is very stable and its existence in the final product would hinder the labeling process. LN3 resin is an extraction resin containing acidic organophosphorus extractants [163], which can be used for the separation of complexing agent (α-HIBA) from Tb and, furthermore, ensures elution of ^161^Tb in a small volume of dilute HCl. The use of LN3 resin, as suggested by Gracheva et al., is used as the last step of ^161^Tb production to avoid the lengthy evaporation step [162], previously needed to obtain the final solution in dilute acid. This procedure cannot replace the evaporation step in the ^177^Lu separation process, however, due to the high distribution coefficient of Lu in dilute acids on LN3 resin [164].

When using the α-HIBA separation system, the separation factors for Lu/Yb (1.55) and Gd/Tb (1.57) are very close [165]. In addition, both radionuclides’ production processes require the recovery of the enriched target material. The elemental analysis of the recycled target material is essential to determine the quality of the material before irradiation.

### 4.3. Labeling Efficiency

Other than their decay properties, another intriguing feature of the radiolanthanides is that they share similar chemical characteristics such as analogous coordination chemistry. They exist mainly in the +3 oxidation state and form eight coordination complexes with smaller ions and nine coordination complexes with larger ions. As a result, they can be linked to a variety of molecular carriers such as small molecules, peptides, proteins and antibodies [166,167,168,169,170].

Lanthanides cations form insoluble hydroxides at pH >6, making the optimal pH for radiolabeling in the range of pH 4-6. An identical labeling procedure can be applied for both ^161^Tb and ^177^Lu thanks to the similar coordination chemistry. The decay products of ^177^Lu and ^161^Tb are Hf^4+^ and Dy^3+^, respectively. Breeman et al. reported that Hf^4+^ does not interfere with Lu binding with the DOTA-chelator [78]. On the other hand, the decay product of ^161^Tb is Dy, which could well interfere with Tb labeling. The radiolabeling yield of ^161^Tb-DOTA has been investigated and it was shown that even after two weeks, the molar activity of the product was sufficient for potential clinical application [162]. Comparison studies have shown that ^161^Tb- and ^177^Lu-DOTATOC (solutions in saline at room temperature) are stable over 24 h in the presence of a stabilizer (L-ascorbic acid).

### 4.4. Production for the Clinical Applications

#### 4.4.1. ^177.^ Lu

Currently, commercial n.c.a. ^177^Lu is produced and supplied to the market by ITG, Germany [20] and several GMP protocols have been developed for the synthesis of ^177^Lu-labeled radiopharmaceuticals. ITG has Marketing Authorization and supplies ^177^Lu under the product name EndolucinBeta. Few studies, using complete automated labeling processes of peptides or antibodies with ^177^Lu, have been reported to date. Syntheses investigated are mainly DOTA-based derivatives [91,92,143,171,172]. Sodium ascorbate solution is used as a buffer to adjust the reaction pH from 4.5-4.9. The volume of the precursor (DOTATOC or PSMA-617) is taken based on the delivered radioactivity. Then, the buffer/precursor solutions were transferred to the reaction vial and heated (90^o^C) for 30 min [171]. In these systems, after the synthesis is carried out, the product from the reaction vial is loaded onto a solid phase extraction (SPE) C18 cartridge to purify the final product from any free activity still in the solution. The radiopharmaceutical is then eluted from the C18 cartridge with an ethanol/water mixture and diluted with 0.9% saline into the final product vial. The final product is eluted through a 0.22 μm filter to ensure sterility. In the development stage, instant thin layer chromatography (ITLC), as well as high pressure liquid chromatography (HPLC), are performed as quality control to determine the radiochemical purity of the product. At this stage, sterility and endotoxin are usually not assessed [91,171,172].

#### 4.4.2. ^161.^ Tb

^161^Tb is not yet commercially available and is not produced based on pharmaceutical legislation in EU-GMP regulations. It was demonstrated that the ^161^TbCl_3_ final product is suitable for pharmaceutical use without requiring further purification and sterilization [162]. Due to the similar chemical characteristics of ^161^Tb and ^177^Lu, the synthesis module protocols commonly used for ^177^Lu synthesis can be directly adapted for the production of ^161^Tb radiopharmaceuticals. Nevertheless, ^161^TbCl_3_ solution has to be validated for GMP use.

Moreover, it is worth mentioning that, due to the low-energy gamma photons which lead to substantial self-absorption, the geometry effect on the activity measurement (see Section 2.1) with dose calibrators for ^161^Tb should be assessed. Table 8 shows that the quality of ^161^Tb is comparable with commercially-available ^177^Lu (EndolucinBeta-ITG) and it is suitable for future clinical applications.

## 5. Conclusions

Radionuclide production is the first step for the preparation of radiopharmaceuticals. To achieve the aim of routine large-scale production, process development of each step in the radionuclide production process is vital. It should be also underlined that radionuclide production for nuclear medical applications is a process under continuous development and optimization.

The success of preclinical studies strongly depends on the availability of the novel radionuclides, as well as the quality thereof. The number of high-intensity intermediate/high-energy and multiple-particle cyclotrons plays a crucial role in the development and the future availability of the novel radionuclides. Furthermore, it is foreseen that in the near future, the sources of nuclear research reactors will not be sufficient due to the wide use of therapeutic β^-^-emitting radionuclides.

^161^Tb and ^44^Sc are novel radionuclides for radiotherapy and PET application, respectively, and seen as a possible improvement to what is currently utilized in a clinical setting. Currently, development of these radionuclides has progressed to the point where the radionuclide precursor reproducibly meets the required quality and the protocols being developed for an automated module for the direct radiolabeling. However, up to date, there is no monograph in the European Pharmacopeia for the preparation of ^161^Tb and ^44^Sc radiopharmaceuticals and there is an urgent need for the definition of the regulation and legislation of the manufacturing thereof, as well as the related quality documentation of the radionuclide precursors. In parallel, more preclinical and clinical comparison studies with ^161^Tb and ^177^Lu will successfully guide the translation of ^161^Tb to the clinics.

## Figures and Tables

**Table 1:** Decay characteristics and applications of radiometals currently used in clinical practice (decay data are taken from [22] and only the main γ-emission lines are shown).

Radionuclide	Half-Life	Main Decay Mode	Eα [MeV]	Eβ_av_ [MeV]	Eγ [KeV]	Iγ [%]	Application
^64^Cu	12.70 h	β^+^		0.278	511 1345.8	(annihil.) 0.48	PET
^67^Ga	9.26 d	γ			93.3 184.6 300.2 393.5 208.9	38.8 21.4 16.6 4.6 2.5	SPECT
^68^Ga	67.71 min	β^+^		0.830	511 1077.3	(annihil.) 3.22	PET
^111^In	2.80 d	γ			245.3 171.3	94.1 90.7	SPECT
^177^Lu	6.65 d	β^−^		0.134	208.4 112.9	10.4 6.2	β^−^-Therapy
^153^Sm	46.50 h	β^−^		0.224	103.2 69.7	29.3 4.7	Bone metastases
^89^Sr	50.53 d	β^−^		0.587			Bone metastases
^223^Ra	11.43 d	α	5.716 5.606 5.539 5.747		269.5 154.2 323.9 144.2	13.9 5.7 3.9 3.3	α-Therapy
^186^Re	3.72 d	β^−^		0.347	137.2	9.5	Bone metastases
^99m^Tc	6.01 h	γ			140.5	89	SPECT
^201^Tl	72.91 h	γ			167.4 135.3	10.0 2.6	SPECT
^90^Y	64.10 h	β^−^		0.934			β^−^-Therapy
^89^Zr	78.41 h	β^+^		0.396	511 909.1	(annihil.) 99	PET

**Table 2:** Comparison of ^44^Sc and ^68^Ga decay characteristics and production routes.

Radionuclide	Half-life	Decay mode	E_γ_ (kev)	Main Production Route	Radionuclidic impurities
^68^Ga		EC (11%)		^68^Ge →^68^Ga generator	^68^Ge ^67^Ga, ^66^Ga,
	67.71 min	β^+^ (88%)	1077 (3%)	^68^Zn(p,n)^68^Ga	
		Eβ^+^_av_: 830 keV			
^44^Sc		EC (5%),		^44^Ti → ^44^Sc generator	^48^Sc, ^44m^Sc
	4.04 h	β^+^ (95%)	1157 (99.9%)	^nat^Ca(p, xn)^44^Sc	^44m^Sc, ^47^Sc, ^48^Sc
		Eβ^+^_av_: 632 keV		^44^Ca(p, n)^44^Sc	^44m^Sc

**Table 3:** Target material used for ^44^Sc and ^68^Ga production, respectively.

^68^Ga	References	^44^Sc	References
Liquid Target		Liquid target	
^68^ZnCl_2_	[93]	Ca(NO_3_)_2_	[94]
^68^Zn(NO_3_)_2_	[42,95,96,97]		
**Solid Target**		**Solid target**	
^68^Zn foil	[98]	^44^CaCO_3_	[99,100]
EP ^68^Zn	[101,102,103]	^44^CaO	[104]
EP: Electroplated			

**Table 4:** Specification for generator-produced ^68^Ga [147].

Final product	^68^GaCl_3_
Appearance	Clear, colorless solution
pH	<2
Radiolabeling Yield	>99%
TLC/HPLC	
Radionuclide Identity (γ-Spectrometry Approx. Half Life)	511 keV 1077 keV
Radionuclidic purity (γ-spectrometry)	>99.9%
Radiochemical Purity (TLC)	>95%
^68^Ge Breakthrough	<0.001%
Bacterial Endotoxins	<175IU/V
Iron	<10ug/GBq, each
Zinc	

**Table 5:** Radionuclidic specifications based on the European Pharmacopoeia for generator and cyclotron-produced ^68^Ga [table adapted from [148].

Final product	Generator-produced ^68^Ga	Cyclotron-produced ^68^Ga
^68^Ga activity	Minimum 99.9%	Minimum 98.0%
^68^Ge activity	Maximum 0.001%	n.a.
^66^Ga and ^67^Ga	n.a.	Maximum 2.0%
Other radioimpurities	n.a.	Maximum 0.1%
n.a. not applicable		

**Table 6:** Comparison of ^177^Lu and ^161^Tb decay characteristics and production routes (data are taken from [22]).

Radionuclide	Half-Life (d)	E_β-av_ (MeV)	E_γ_ (kev)	Auger/Conversion e^-^		Production Route
^177^Lu	6.64 [152]	134	208 (10.4%)	Auger L	6.3 (8.7%)	^176^Lu(n,γ)^177^Lu
			113 (6.4%)	CE K	47.6 (5.1%)	^176^Yb(n,γ)^177^Yb →^177^Lu
			321 (0.219%)	CE L	101.7 (6.8%)	
^161^Tb	6.96 [153]	154	26 (23.2%)	CE K	3.4 (17.5%)	^160^Gd(n,γ)^161^Gd →^161^Tb
			29 (0.0365%)	CE L	16.6 (41%)	
			44 (0.060%)	Auger L	5.2 (87.9%)	

**Table 7:** Comparison of neutron activation products of ^176^Lu, ^176^Yb, and ^160^Gd.

Target	% Natural Abundance	% Available Enrichment	Cross Section σ (Barn)	Activation Product	Decay Mode	T_1/2_	Decay Product	Specific Activity (TBq/mg)
^176^Lu_2_O_3_	2.59	84.6	2.8	^177m^Lu	β, γ, IT	160.4 d	^177^Hf (78.6%) ^177^Lu (21.4%)→	3.9 TBq/mg
			2090	^177^Lu	β, γ	6.65 d	^177^Hf	
^176^Yb_2_O_3_	12.76	>99.6	2.85	^177^Yb	β, γ	1.9 h	^177^Lu	740-1110 GBq/mg
^160^Gd_2_O_3_	21.86	98.2	1.5	^161^Gd	β, γ	3.66 m	^161^Tb	to be optimized

**Table 8:** Comparison of the specification for ^177^LuCl_3_ and ^161^TbCl_3_ [162,173]_._

Test	^177^LuCl_3_ (EndolucinBeta)	^161^TbCl_3_
Specific activity (Dose calibrator)	36–44 GBq/mL at ART	11–21 GBq/mL
Appearance	Clear, colorless	Clear, colorless
pH (pH indicator strip)	1–2	1–2
Radiolabeling yield	>99.0%	>99.0%
TLC/HPLC (Based on radiolabeling with ^177^Lu or ^161^Tb of DOTA-derivate, molar ratio 1:4)		
Identity (γ-spectrometry)	113 keV 208 keV	48.9 keV 74.6 keV
Radionuclidic purity (γ-spectrometry)	^175^Yb ≤ 0.1%	^160^Tb ≤ 0.007%
Radiochemical purity (radio-TLC)	>99.0%	>99.0%
Bacterial endotoxins	≤175 IU/V	

ART: Activity reference time.
